# Non-elective colectomy for diverticulitis in the U.S.: a retrospective comparison of robotic, laparoscopic, and open approaches

**DOI:** 10.1186/s13017-026-00700-3

**Published:** 2026-05-14

**Authors:** Leon Naar, Prajakta H. Waghmare, I-Fan Shih, Sherry M. Wren

**Affiliations:** 1 Department of Surgery, Stanford University School of Medicine, Stanford, CA USA; 2 Intuitive Surgical, Sunnyvale, CA USA; 3 Palo Alto Veterans Health Care System, Palo Alto, CA USA; 4 Stanford University School of Medicine, 3801 Miranda Avenue G112 PACAHCS, Palo Alto, CA 94304 USA

**Keywords:** Acute diverticulitis, Emergency surgery, Robotic, Minimally invasive

## Abstract

**Background:**

Minimally invasive techniques are increasingly used in emergency surgery, including in acute diverticulitis. While studies have shown that laparoscopic colectomy in the emergent/urgent management of acute diverticulitis has better outcomes compared to open surgery, data on robotic surgery outcomes remain limited. This study aims to compare the outcomes of patients with acute diverticulitis requiring non-elective operations across open, laparoscopic, and robotic surgical approaches.

**Methods:**

A retrospective analysis from the Premier healthcare database (2019–2023) was conducted, including patients undergoing non-elective left or sigmoid colectomy for acute diverticulitis. Emergency surgery was defined as colectomy performed within 48 h from admission; urgent surgery was defined as colectomy performed between 2 and 12 days after admission. Our primary outcome was conversion to open surgery. Secondary outcomes included anastomotic leak, stoma creation during the index operation, stoma creation within 30 days from surgery, hospital length of stay, readmission and reoperation rates. Propensity Score Matching (PSM) 1:1 was applied to adjust for patient demographics, comorbidities, disease severity and hospital/surgeon characteristics.

**Results:**

A total of 28,456 patients were included in the analysis [17,934 (63%) emergent; 10,522 (37%) urgent surgery]. Open surgery was the predominant modality (77.6% emergent; 63.2% urgent) with robotic surgery use increasing threefold during the study period. On univariate analysis, emergent surgery was more common in patients requiring ICU admission and patients with associated peritoneal abscess. Robotic surgery was more common in younger patients, with fewer comorbidities, as well as in urban mid-size institutions. After PSM, robotic surgery was associated with lower rates of conversion to open surgery compared to laparoscopic surgery (emergent setting: 10.2% vs 22.6%, *p*-value < 0.001; urgent setting: 13% vs 27.8%, *p*-value < 0.001). Patients undergoing robotic surgery also had lower rates of stoma creation at index operation, and shorter hospital stay compared to laparoscopic surgery (emergent and urgent operations). In emergent colectomies only, robotic surgery was associated with a 29% relative-risk reduction of anastomotic leaks [laparoscopic 9.6% vs. 6.8%, *p*-value = 0.029].

**Conclusions:**

The robotic platform is expanding rapidly within the emergency general surgery patient population. Robotic colectomy for non-elective diverticulitis is a safe and feasible option with improved post-operative outcomes when compared to laparoscopic surgery.

**Supplementary Information:**

The online version contains supplementary material available at 10.1186/s13017-026-00700-3.

## Background

Diverticulitis is one of the most common gastrointestinal disease diagnoses, with an annual treatment cost that exceeds 2.5 billion dollars in the US [1]. Over the past decades, an increase in the incidence of patients diagnosed with acute diverticulitis has been observed, leading to an increase in the number of patients requiring non-elective surgery [2, 3]. Approximately 10–20% of all patients admitted with acute diverticulitis will fail non-operative management and require an emergent or urgent surgical intervention [4–7].

Multiple studies have examined the safety and feasibility of robotic surgery in elective colectomies [8]. The benefits in this setting are clear: reduced pain, lower rates of postoperative ileus, and shorter length of stay. However, the emergency general surgery patient population is a unique subset of patients with a disproportionately higher rate of morbidity and mortality [9]. Traditionally, the focus in acute care surgery has been survival-based, with open approaches predominating and delayed adoption of minimally invasive surgery for more complex procedures [10]. However, a shift towards minimally invasive approaches has been observed, with recent studies showing better outcomes of laparoscopic colectomy to open surgery in emergent/urgent settings for acute diverticulitis [8].

Over the past decade, the use of robotic surgery has also increased in abdominal emergencies [11]. Nevertheless, the majority of the published studies investigating the use of robotic surgery in emergent or urgent colorectal surgery have been small, single-institution studies [2, 12]. In addition, the rapid expansion of robotic platform use in the acute care setting has led to significant financial concerns given the heterogeneity of outcomes reported across studies and clinical settings [13]. Moreover, the steep learning curves together with the documented volume-outcome association have also raised patient safety concerns regarding the use of robotic surgery in the acute setting [6, 14].

With the expansion of the robotic platform in the emergency general surgery patient population, urgent research is required to identify the optimal balance between patient outcomes and the cost justification for robotic surgery. In the present study, we sought to compare outcomes among patients with acute diverticulitis requiring an emergent/urgent operation during the index admission in association with the surgical modality used (open, laparoscopic, robotic surgery). We hypothesized that robotic surgery would have a similar outcomes profile to laparoscopic surgery and that both would be associated with improved outcomes than open surgery.

## Methods

### Data source

The Premier Healthcare dataset (PHD) is a large, US hospital-based, Health Insurance Portability and Accountability Act (HIPAA)-compliant database containing inpatient and outpatient data from diverse hospitals and healthcare systems [15]. It includes more than 135 million inpatient admissions, representing 25% of annual US inpatient admissions. This study was deemed exempt from informed consent and institutional review board (IRB) approval. This study is reported in accordance with the STROBE checklist for cohort studies [16].

### Patient population

**Fig. 1:**
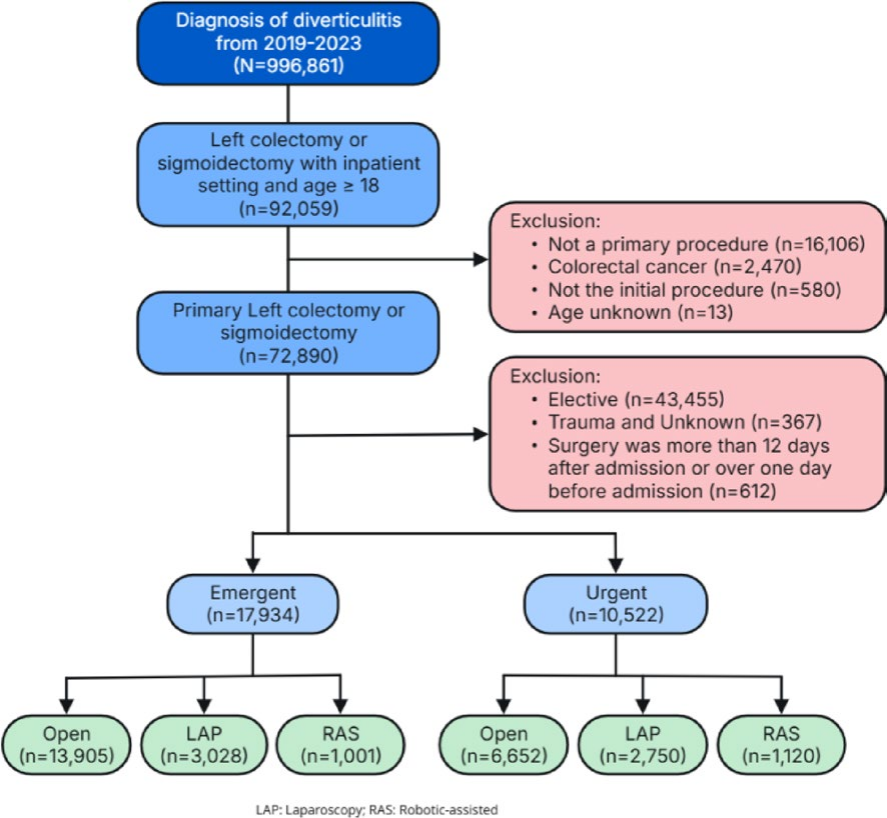
Flowchart of study patient selection

Adult patients admitted for the management of acute diverticulitis between January 1, 2019, and December 31st, 2023, were identified through International Classification of Diseases, 10th Revision (ICD-10) and included in the analysis. Among these, ICD-10 Procedure Coding System (PCS) and Current Procedural Terminology (CPT) codes were used to identify patients who underwent left or sigmoid colectomy during the index admission (Supplementary Table 1). Patients were excluded if they did not have a left or sigmoid colectomy as the primary procedure, had a diagnosis of colorectal cancer, or if their age was missing (Fig. 1).

### Definition of emergent and urgent surgery

Emergent surgery was defined as a colectomy that occurred within 48 h during the index admission for acute diverticulitis [17]. Urgent surgery was defined as a colectomy for acute diverticulitis that occurred during the index admission for the acute diverticulitis, but after 48 h and before day 12 from admission.

### Covariates

Patient socio-demographic characteristics analyzed included age, gender, race, ethnicity, and insurance type (e.g. Medicare, Medicaid, commercial, or others). The year of admission and clinical characteristics, including body mass index (BMI), smoking status, Charlson Comorbidity Index (CCI) score, need for ICU admission, presence of peritoneal abscess, and need for lysis of adhesions, were captured. Hospital characteristics included geographical region (Midwest, Northwest, South, or West), hospital type (teaching vs. non-teaching), location (rural vs. urban), and bed size (< 200, 200–499, or > 500). Hospital volume was calculated annually as the total number of emergent, urgent, or elective left or sigmoid colectomies for diverticulitis and categorized into tertiles: low (≤ 67), medium (68–140), and high (≥ 141). Surgeon volume was defined as the number of left or sigmoid colectomies performed by the operating surgeon in the 12 months prior to the surgery date, specific to the surgical approach (open, laparoscopic or robotic surgery) and categorized into tertiles: low (≤ 3), medium [4–10] and high (≥ 11). To stratify for different disease severity, we used the presence of peritoneal abscess and need for ICU admission occurring prior to or within one day after surgery.

### Outcome measures

The primary outcome measure was conversion to open surgery. Additional secondary outcomes assessed included perioperative complications within 30 days postoperative, including anastomotic leak, ileus, bleeding/need for blood transfusions, surgical site infections (SSI), sepsis, and bowel obstruction (Supplementary Table 1). Stoma creation (colostomy or ileostomy) during the index operation and within 30 days post-discharge were also evaluated. Additionally, hospital resource utilization outcomes, including operating room (OR) time, hospital length of stay, and 30-day readmission and reoperation rates, were assessed. Total costs were derived from hospital cost accounting data and reflect fixed and variable costs, including supplies, labor, and equipment depreciation. Costs were assessed for the index hospitalization (admission through discharge) and for the index hospitalization plus all costs incurred within 30 days post-discharge. All costs were reported in 2023 US dollars.

### Statistical analysis

In bivariate analysis, categorical variables were compared using Pearson’s chi-square test to assess for baseline differences. Continuous variables were compared using two sample t-test. To balance covariates among OS, LS and RS cohorts, propensity-score matching was performed. Our main analysis was on patients undergoing an emergent surgery. Propensity scores were estimated via logistic regression using the covariates mentioned above. Matching was done in pairs (Open-Laparoscopic, Open-Robotic, Laparoscopic-Robotic) using nearest neighbor 1:1 matching with a caliper of 0.1. Post-matching balance was evaluated using standardized mean differences (SMD), with values < 0.01 indicating adequate balance. Outcome comparisons across cohorts were conducted using two sample t-test for continuous variables and Pearson’s chi-square test for categorical variables. A two-tailed *p* value of < 0.05 was considered statistically significant. We also conducted a secondary analysis looking into patients undergoing urgent surgery, as well as a predetermined multivariable regression analysis to identify risk factors for the creation of an ostomy during the index operation. All statistical analyses were performed using R version 4.4.2.

## Results

**Fig. 2:**
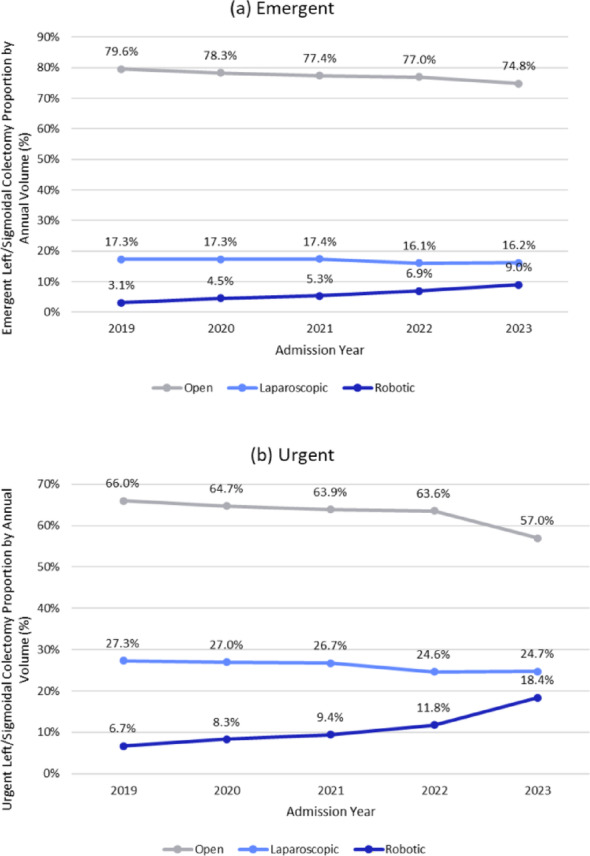
Trends comparing Open, Laparoscopic and Robotic-assisted colectomy by year

Of 996,861 patients diagnosed with acute diverticulitis between 2019 and 2023, 72,890 underwent a left or sigmoid colectomy (Fig. 1). Of these, 17,934 met our definition for emergent operation and 10,522 were classified as urgent cases. Open surgery was the predominant modality, accounting for 77.6% of emergent and 63.2% of urgent procedures. However, we observed a shift in surgical modality use over time. In emergent cases, robotic surgery increased from 3.1% in 2019 to 9.0% in 2023, at the expense primarily of open surgery (declined from 79.6 to 74.8%), whereas laparoscopic surgery rates remained stable (Fig. 2a). The increase in robotic surgery use was more pronounced in urgent cases, climbing from 6.7 to 18.4%, while open surgery decreased from 66.0 to 57.0%, and laparoscopic surgery dropped from 27.3 to 24.7% (Fig. 2b).

### Patient, hospital and surgeon characteristics

The majority of patients were > 65 years old (45.1%), non-Hispanic (79.5%), White (83.1%), and received care in urban, non-academic centers. Table 1 summarizes the socio-demographic and clinical characteristics of emergency and urgent surgery patients included in our analysis. On univariate analysis, patients requiring emergent surgery were slightly older, more likely to be male, less comorbid and not obese compared to patients undergoing urgent surgery (Table 1). Patients requiring emergent surgery were more likely to have an associated peritoneal abscess and more likely to require an ICU admission, indicating higher disease severity at presentation.

**Table 1:** Baseline Patient Characteristics in Emergency and Urgent

Characteristics	Emergent (N = 17,934)	Urgent (N = 10,522)	*p*-value
Age continuous			** < 0.001**
Mean (SD)	62.0 (14.4)	61.2 (14.6)	
Median (Q1, Q3)	63 (52, 73)	62 (51, 72)	
Age category, n (%)			** < 0.001**
18–44 years	2,370 (13.2)	1,575 (15.0)	
45–64 years	7,335 (40.9)	4,333 (41.2)	
65 + years	8,229 (45.9)	4,614 (43.9)	
Gender, n (%)			** < 0.001**
Female	9,490 (52.9)	5,852 (55.6)	
Male	8,444 (47.1)	4,670 (44.4)	
Hispanic Ethnicity, n (%)			** < 0.001**
Yes	1,688 (9.4)	1,283 (12.2)	
No	14,479 (80.7)	8,145 (77.4)	
Unknown	1,767 (9.9)	1,094 (10.4)	
Race, n (%)			** < 0.001**
White	15,089 (84.1)	8,548 (81.2)	
Black	1,275 (7.1)	969 (9.2)	
Other	993 (5.5)	668 (6.3)	
Unknown	577 (3.2)	337 (3.2)	
Charlson Comorbidity Index, n (%)			**0.035**
CCI = 0	8,533 (47.6)	4,857 (46.2)	
CCI = 1	4,239 (23.6)	2,609 (24.8)	
CCI > = 2	5,162 (28.8)	3,056 (29.0)	
BMI category, n (%)			** < 0.001**
Not Obese	13,134 (73.2)	7,253 (68.9)	
Obese	4,800 (26.8)	3,269 (31.1)	
Current or recurrent smoker, n (%)	8,133 (45.3)	5,092 (48.4)	** < 0.001**
Lysis of adhesions, n (%)	2,317 (12.9)	1,952 (18.6)	** < 0.001**
Peritoneal abscess, n (%)	9,206 (51.3)	3,474 (33.0)	** < 0.001**
ICU admission before OR, day of surgery, or the day after surgery, n (%)	5,413 (30.2)	2,097 (19.9)	** < 0.001**
Year of admission, n (%)			0.13
2019	4,012 (22.4)	2,300 (21.9)	
2020	3,546 (19.8)	2,164 (20.6)	
2021	3,689 (20.6)	2,147 (20.4)	
2022	3,642 (20.3)	2,050 (19.5)	
2023	3,045 (17.0)	1,861 (17.7)	
Hospital location, n (%)			** < 0.001**
Urban	15,571 (86.8)	9,347 (88.8)	
Rural	2,363 (13.2)	1,175 (11.2)	
Payor, n (%)			**0.006**
Medicare	8,358 (46.6)	4,755 (45.2)	
Commercial	6,146 (34.3)	3,644 (34.6)	
Medicaid	1,976 (11.0)	1,155 (11.0)	
Other	1,454 (8.1)	968 (9.2)	
Provider Region, n (%)			** < 0.001**
Northeast	2,842 (15.8)	1,665 (15.8)	
Midwest	3,961 (22.1)	1,997 (19.0)	
South	8,380 (46.7)	5,270 (50.1)	
West	2,751 (15.3)	1,590 (15.1)	
Hospital number of beds, n (%)			** < 0.001**
0–199 beds	4,562 (25.4)	2,427 (23.1)	
200–499 beds	8,670 (48.3)	5,228 (49.7)	
500 + beds	4,702 (26.2)	2,867 (27.2)	
Academic institution, n (%)	7,929 (44.2)	4,601 (43.7)	0.43
Hospital volume, n (%)			** < 0.001**
Low Volume (0–67)	8,080 (45.1)	4,297 (40.8)	
Medium Volume (68–140)	5,644 (31.5)	3,727 (35.4)	
High Volume (141–425)	4,210 (23.5)	2,498 (23.7)	
Surgeon volume, n (%)			** < 0.001**
Low Volume (0–3)	8,415 (46.9)	4,279 (40.7)	
Medium Volume (4–10)	6,311 (35.2)	3,653 (34.7)	
High Volume (11–167)	3,208 (17.9)	2,590 (24.6)	

Abbreviations: SD: Standard Deviation; CCI: Charlson Comorbidity Index; BMI: Body Mass Index; ICU: Intensive Care Unit

Supplementary Table 2 summarizes the baseline patient socio-demographic and clinical characteristic by surgical modality. Across both emergent and urgent procedures, robotic surgery was more frequently used in younger (< 65 years old) and less comorbid patients as well as patients with lower rates of peritoneal abscesses. The rates of robotic surgery use were lower among patients requiring ICU admission. Robotic use was more prevalent in patients with commercial insurance, and in urban and mid-size institutions (200–499 beds).

### Robotic surgery outcomes in emergent surgery

After PSM matching all the cohorts were balanced at SMD < 0.01. SMD plots before and after matching are shown in Supplementary Fig. 1. Table 2 summarizes the results after propensity score matching comparing open, laparoscopic, and robotic emergent surgery. Conversion to open surgery was significantly lower in the robotic, compared to the laparoscopic surgery group (10.2% vs 22.6%, *p*-value < 0.001). Any minimally invasive approach had longer operative time when compared to open surgery with robotic surgery being significantly longer than laparoscopic surgery (mean 254.2 vs 198.8 min, *p*-value < 0.001). With regards to post-operative length of stay, robotic colectomy was associated with lower length of stay than open (mean 5.8 vs 8 days, *p*-value < 0.001) and laparoscopic colectomy (mean 5.9 vs 6.5 days, *p*-value = 0.014). While there was no difference between open and laparoscopic surgery, anastomotic leaks were significantly lower in robotic surgery (robotic vs open: 7% vs 9.7%, *p*-value = 0.034; robotic vs laparoscopic: 6.8% vs 9.6%, *p*-value = 0.029). Minimally invasive approaches had lower rates of post-operative ileus, with the incidence after robotic surgery being significantly lower than that after laparoscopic surgery (10.9% vs 15.7%, *p*-value = 0.002). While there was no difference observed between robotic and laparoscopic surgery, patients undergoing a minimally invasive approach, when compared to open surgery, were more likely to be discharged home, have lower in-hospital mortality, and also less likely to be readmitted within 30 days from the operation. Lastly, stoma creation during the index operation was significantly lower in robotic vs laparoscopic surgery (29.5% vs 43.1%, *p*-value < 0.001) without any difference in reoperations or need for diversion within 30 days from the operation. With regards to healthcare cost, no difference was found between open and laparoscopic surgery. However, the cost associated with robotic surgery was significantly higher than open (index cost mean $30,342.1 vs. $27,433.1, *p*-value = 0.001) and laparoscopic surgery (index cost mean $30,683.3 vs. $27,243.8, *p*-value < 0.001).

**Table 2:** Propensity Score Matched Outcomes for Emergent Colectomy Comparing Open (OS), Laparoscopic (LS), and Robotic (RS) surgery

Outcomes	OS vs LS			OS vs RS			LS vs RS		
	OS (N = 3023)	LS (N = 3023)	*p*-value	OS (N = 984)	RS (N = 984)	*p*-value	LS (N = 941)	RS (N = 941)	*p*-value
Conversion, n (%)	0 (0.0)	892 (29.5)	**NA**	0 (0.0)	95 (9.7)	**NA**	213 (22.6)	96 (10.2)	** < 0.001**
OR time			** < 0.001**			** < 0.001**			** < 0.001**
Mean (SD)	176.6 (193.2)	204.4 (104.8)		170.8 (82.4)	253.8 (277.3)		198.8 (103.1)	254.2 (282.6)	
Median (Q1, Q3)	165 (122, 210)	195 (150, 255)		165 (124, 210)	240 (180, 300)		192 (150, 245)	240 (180, 300)	
Length of stay			** < 0.001**			** < 0.001**			**0.002**
Mean (SD)	9.4 (7.5)	8.3 (6.4)		8.7 (6.2)	6.4 (5.4)		7.3 (4.7)	6.6 (5.5)	
Median (Q1, Q3)	8 (6, 11)	7 (5, 10)		7 (6, 10)	5 (3, 8)		6 (5, 9)	5 (3, 8)	
Post-operative length of stay			** < 0.001**			** < 0.001**			**0.014**
Mean (SD)	8.8 (7.5)	7.5 (6.4)		8.0 (6.2)	5.8 (5.3)		6.5 (4.6)	5.9 (5.4)	
Median (Q1, Q3)	7 (5, 10)	6 (4, 9)		7 (5, 9)	4 (3, 7)		5 (4, 8)	4 (3, 7)	
Blood transfusion: 30d, n (%)	278 (9.2)	250 (8.3)	0.20	81 (8.2)	57 (5.8)	**0.034**	59 (6.3)	58 (6.2)	0.92
Bleeding: 30d, n (%)	397 (13.1)	334 (11.0)	**0.013**	143 (14.5)	92 (9.3)	** < 0.001**	99 (10.5)	93 (9.9)	0.65
Anastomotic leak: 30d, n (%)	261 (8.6)	268 (8.9)	0.75	95 (9.7)	69 (7.0)	**0.034**	90 (9.6)	64 (6.8)	**0.029**
Ileus: 30d, n (%)	638 (21.1)	534 (17.7)	** < 0.001**	178 (18.1)	109 (11.1)	** < 0.001**	148 (15.7)	103 (10.9)	**0.002**
Surgical site infections: 30d, n (%)	294 (9.7)	225 (7.4)	**0.002**	94 (9.6)	55 (5.6)	** < 0.001**	65 (6.9)	53 (5.6)	0.25
Sepsis: 30d, n (%)	445 (14.7)	346 (11.4)	** < 0.001**	150 (15.2)	70 (7.1)	** < 0.001**	81 (8.6)	71 (7.5)	0.40
Bowel obstruction: 30d, n (%)	153 (5.1)	118 (3.9)	**0.030**	62 (6.3)	36 (3.7)	**0.007**	43 (4.6)	34 (3.6)	0.29
ICU admission post-operative, n (%)	350 (11.6)	308 (10.2)	0.083	63 (6.4)	48 (4.9)	0.14	45 (4.8)	49 (5.2)	0.67
Readmission: 30 days, n (%)	327 (10.8)	281 (9.3)	**0.049**	128 (13.0)	89 (9.0)	**0.005**	79 (8.4)	87 (9.2)	0.52
Reoperation: 30d, n (%)	158 (5.2)	126 (4.2)	0.052	40 (4.1)	33 (3.4)	0.40	21 (2.2)	32 (3.4)	0.13
Discharge to home, n (%)	2,440 (80.7)	2,583 (85.4)	** < 0.001**	826 (83.9)	904 (91.9)	** < 0.001**	859 (91.3)	862 (91.6)	0.80
In-hospital mortality rate, n (%)	70 (2.3)	48 (1.6)	**0.041**	18 (1.8)	3 (0.3)	** < 0.001**	7 (0.7)	3 (0.3)	0.20
Stoma during surgery, n (%)	2,092 (69.2)	1,614 (53.4)	** < 0.001**	637 (64.7)	281 (28.6)	** < 0.001**	406 (43.1)	278 (29.5)	** < 0.001**
Stoma: 30d post-operative, n (%)	76 (2.5)	64 (2.1)	0.30	17 (1.7)	13 (1.3)	0.46	9 (1.0)	13 (1.4)	0.39
Index cost, USD			0.3			**0.001**			** < 0.001**
Mean (SD)	32,211.0 (33,732.4)	31,415.6 (25,142.5)		27,433.1 (20,581.4)	30,342.1 (19,148.3)		27,243.8 (16,677.7)	30,683.3 (19,457.1)	
Median (Q1, Q3)	23,986 (18,121, 35,056)	24,911 (18,682, 35,354)		22,447 (17,185, 30,850)	25,342 (19,343, 34,394)		23,259 (17,897, 30,939)	25,737 (19,554, 34,715)	
Index and 30-day cost, USD			0.17			**0.028**			** < 0.001**
Mean (SD)	34,506.1 (35,447.2)	33,402.1 (26,998.2)		29,941.2 (22,891.6)	32,154.9 (21,850.6)		28,976.2 (18,672.2)	32,532.1 (22,184.1)	
Median (Q1, Q3)	25,510 (18,618, 37,896)	25,870 (19,326, 37,397)		23,316 (17,642, 34,047)	26,341 (19,663, 36,320)		24,075 (18,454, 32,457)	26,660 (19,804, 36,732)	

Abbreviations: OS: Open Surgery; LS: Laparoscopic Surgery; RS: Robotic Surgery; OR: Operating Room; SD: Standard Deviation; ICU: Intensive Care Unit, USD: United States Dollar

### Secondary analysis: urgent surgery

The results of the secondary analysis looking into urgent only colectomies are shown in Table 3. After PSM matching all the cohorts were balanced at SMD < 0.01. SMD plots before and after matching are shown in Supplementary Fig. 2. The results of our main analysis were largely maintained with the exception of anastomotic leaks, where no significant difference between laparoscopic and robotic surgery was detected when looking into urgent colectomies. Similar to the primary analysis, patients undergoing an urgent robotic colectomy were less likely to require conversion to open (robotic vs laparoscopic surgery: 13% vs 27.8%, *p*-value < 0.001).

**Table 3:** Propensity Score Matched Outcomes for Urgent Colectomy Comparing Open (OS), Laparoscopic (LS), and Robotic (RS) surgery

Outcomes	OS vs LS			OS vs RS			LS vs RS		
	OS (N = 2722)	LS (N = 2722)	*p*-value	OS (N = 1085)	RS (N = 1085)	*p*-value	LS (N = 1066)	RS (N = 1066)	*p*-value
Conversion, n (%)	0 (0.0)	796 (29.2)	**NA**	0 (0.0)	141 (13.0)	**NA**	296 (27.8)	139 (13.0)	** < 0.001**
Operating Room time			**0.004**			** < 0.001**			** < 0.001**
Mean (SD)	208.8 (426.3)	233.5 (119.0)		192.4 (99.4)	291.4 (447.5)		220.3 (120.3)	304.9 (610.4)	
Median (Q1, Q3)	185 (150, 240)	223 (165, 291)		180 (140, 240)	270 (201, 330)		210 (150, 270)	270 (199, 330)	
Length of stay			** < 0.001**			** < 0.001**			0.070
Mean (SD)	14.0 (7.0)	13.1 (7.0)		14.0 (8.6)	12.3 (7.7)		12.8 (6.5)	12.3 (7.7)	
Median (Q1, Q3)	12 (10, 16)	11 (9, 15)		12 (10, 16)	11 (8, 14)		11 (9, 15)	11 (8, 14)	
Post-operative Length of stay			** < 0.001**			** < 0.001**			**0.002**
Mean (SD)	8.8 (6.5)	7.7 (6.4)		8.9 (8.3)	6.7 (7.0)		7.5 (5.9)	6.7 (7.1)	
Median (Q1, Q3)	7 (5, 10)	6 (4, 9)		7 (5, 10)	5 (3, 7)		6 (4, 9)	5 (3, 8)	
Blood transfusion: 30d, n (%)	311 (11.4)	309 (11.4)	0.93	126 (11.6)	116 (10.7)	0.50	112 (10.5)	118 (11.1)	0.68
Bleeding: 30d, n (%)	402 (14.8)	384 (14.1)	0.49	171 (15.8)	153 (14.1)	0.28	160 (15.0)	153 (14.4)	0.62
Anastomotic leak: 30d, n (%)	293 (10.8)	274 (10.1)	0.40	141 (13.0)	124 (11.4)	0.27	107 (10.0)	124 (11.6)	0.33
Ileus: 30d, n (%)	552 (20.3)	491 (18.0)	**0.036**	234 (21.6)	145 (13.4)	** < 0.001**	188 (17.6)	143 (13.4)	**0.006**
Surgical site infections: 30d, n (%)	329 (12.1)	279 (10.2)	**0.031**	124 (11.4)	104 (9.6)	0.16	105 (9.8)	103 (9.7)	0.88
Sepsis: 30d, n (%)	399 (14.7)	338 (12.4)	**0.016**	162 (14.9)	108 (10.0)	** < 0.001**	134 (12.6)	105 (9.8)	0.065
Bowel obstruction: 30d, n (%)	135 (5.0)	145 (5.3)	0.54	48 (4.4)	56 (5.2)	0.42	54 (5.1)	53 (5.0)	0.92
ICU admission post-operative, n (%)	217 (8.0)	185 (6.8)	0.10	81 (7.5)	61 (5.6)	0.083	64 (6.0)	60 (5.6)	0.71
Readmission: 30d, n (%)	307 (11.3)	303 (11.1)	0.86	132 (12.2)	126 (11.6)	0.69	108 (10.1)	123 (11.5)	0.27
Reoperation: 30d, n (%)	113 (4.2)	112 (4.1)	0.95	53 (4.9)	47 (4.3)	0.54	38 (3.6)	46 (4.3)	0.37
Discharge to home, n (%)	2,186 (80.3)	2,278 (83.7)	**0.001**	860 (79.3)	922 (85.0)	** < 0.001**	886 (83.1)	905 (84.9)	0.19
In-hospital Mortality rate, n (%)	60 (2.2)	40 (1.5)	**0.044**	24 (2.2)	10 (0.9)	**0.016**	13 (1.2)	11 (1.0)	0.68
Stoma during surgery, n (%)	1,608 (59.1)	1,138 (41.8)	** < 0.001**	597 (55.0)	337 (31.1)	** < 0.001**	420 (39.4)	328 (30.8)	** < 0.001**
Stoma: 30d after surgery, n (%)	54 (2.0)	48 (1.8)	0.55	22 (2.0)	21 (1.9)	0.88	15 (1.4)	21 (2.0)	0.31
Index cost, USD			0.34			**0.006**			**0.02**
Mean (SD)	40,624.2 (30,154.5)	41,428.2 (32,585.1)		38,877.1 (25,425.3)	41,889.8 (26,072.0)		39,136.0 (29,098.0)	41,925.4 (26,036.0)	
Median (Q1, Q3)	33,374 (25,324, 45,652)	33,996 (25,891, 46,802)		32,391 (24,230, 44,601)	36,266 (27,167, 50,243)		32,687 (25,125, 43,469)	36,480 (27,266, 50,259)	
Index and 30-day cost, USD			0.22			**0.032**			**0.04**
Mean (SD)	43,057.8 (32,266.4)	44,200.8 (36,123.5)		41,458.1 (27,895.8)	44,085.2 (29,258.0)		41,520.6 (31,397.9)	44,225.1 (29,343.2)	
Median (Q1, Q3)	35,121 (26,282, 48,864)	35,345 (26,747, 49,518)		34,242 (25,199, 48,725)	37,762 (27,691, 52,338)		33,613 (25,899, 46,141)	37,878 (27,833, 52,569)	

Abbreviations: OS: Open Surgery; LS: Laparoscopic Surgery; RS: Robotic Surgery; OR: Operating Room; SD: Standard Deviation; ICU: Intensive Care Unit, USD: United States Dollar

### Predictors of stoma creation at index operation

**Fig. 3:**
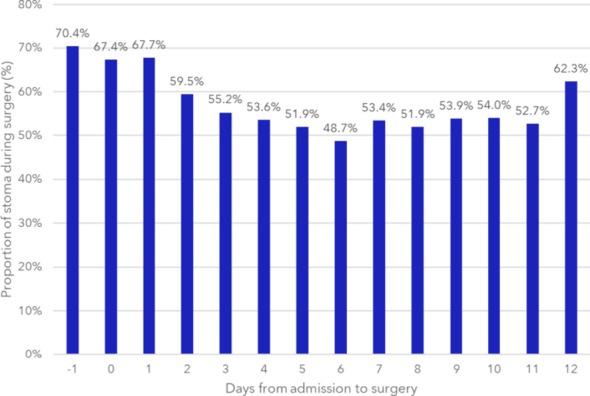
Rates of stoma creation during index surgery in correlation with days from admission to surgery

**Fig. 4:**
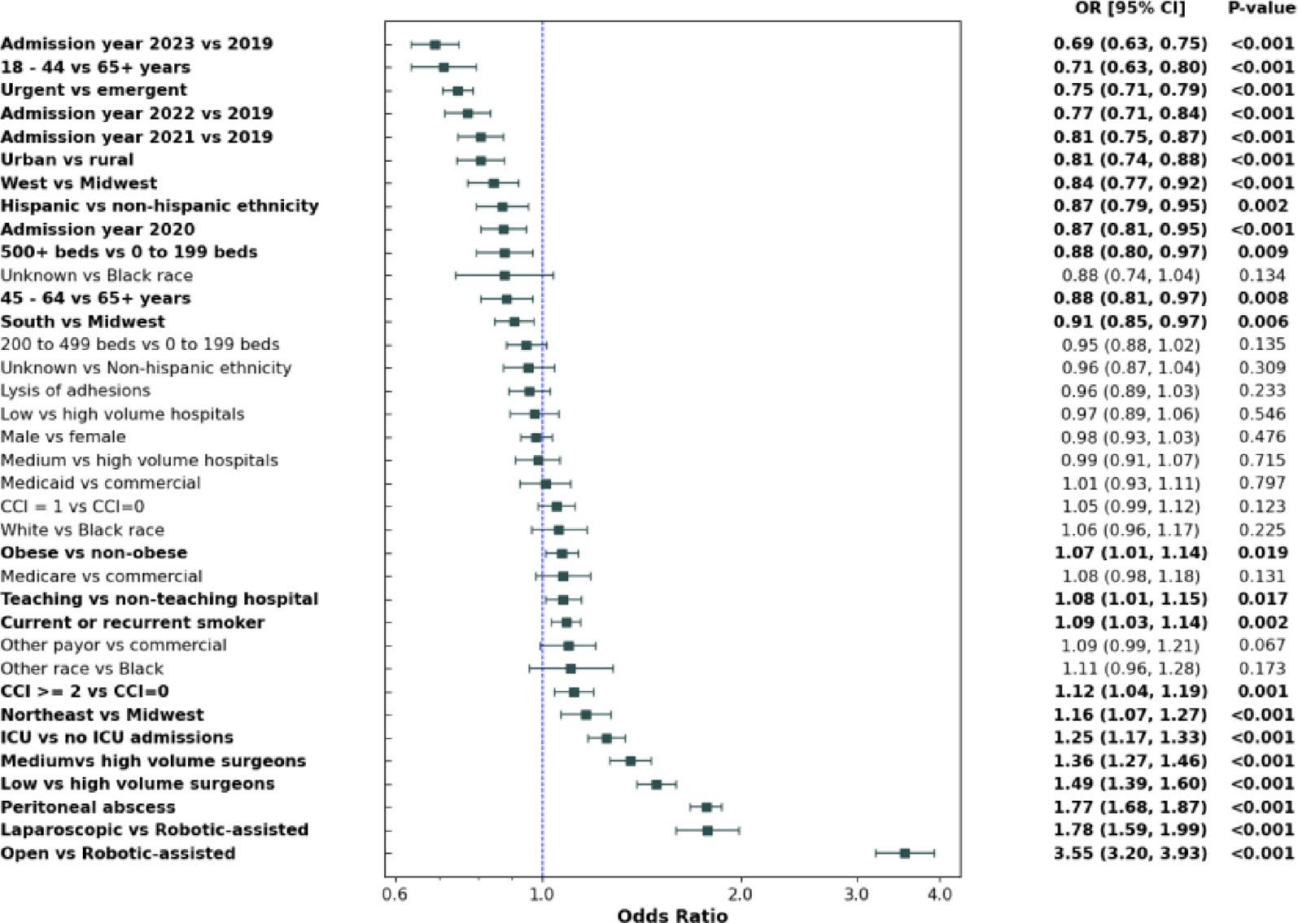
Multivariable regression analysis showing the risk factors for ostomy creation during the index operation

A decreasing trend in stoma creation was observed as the interval from admission to surgery increased (Fig. 3). When looking into the predictors for stoma creation during the index operation, urgent colectomies had lower odds of stoma creation than emergent ones (aOR: 0.75, 95% CI 0.71–0.79) (Fig. 4). In terms of surgical modality, both laparoscopic (aOR: 1.78; 95% CI 1.59–1.99) and open surgery (aOR: 3.55; 95% CI 3.20–3.93) were associated with higher odds of stoma creation at the index operation compared to robotic surgery. Additional factors that independently increased stoma creation included the presence of a peritoneal abscess, operations performed by low- or medium-volume surgeons, ICU admission before surgery, CCI ≥ 2, current or recurrent smoking status, surgeries performed in teaching hospitals, and obesity.

## Discussion

In this retrospective analysis of a large nationwide database, we demonstrated that robotic surgery is a safe and feasible modality in patients undergoing emergent or urgent colectomy for acute diverticulitis. We found a consistent association between robotic surgery and lower rates of conversion to open surgery, lower rates of stoma creation, and quicker post-operative recovery with shorter length of hospital stay. In emergent colectomies, lower rates of anastomotic leaks and ileus were also observed compared to laparoscopic approaches. To our knowledge, ours is the first study to demonstrate that robotic surgery is an advantageous surgical modality for the emergent/urgent management of acute diverticulitis.

The observed rates of open colectomies for patients with acute diverticulitis were higher than those reported in the literature [8]. This can likely be explained by the addition of emergent colectomies in our cohort. During our study period, we observed a threefold increase in robotic colectomy rates for acute diverticulitis, accompanied by a primary decrease in open surgery rates. Keller et al. found that fellowship-trained colorectal surgeons were more likely to perform non-elective cases using minimally invasive techniques than general surgeons [18]. Our study was not able to evaluate the specialty training of the operating surgeon; however, the observed decrease in the rates of open surgery could be the result of increased specialization and robotic training during residency and fellowship.

In a study by Juo et al. on elective colectomies for diverticulitis, the authors did not identify a benefit of robotic over laparoscopic surgery in terms of conversion to open surgery or ostomy creation, whereas hospitalization costs were significantly higher in the robotic group [19]. In a separate randomized controlled trial looking into robotic compared to laparoscopic surgery for elective oncologic right colectomies, again no benefit was seen with the use of robotic surgery in terms of conversion to open surgery, postoperative morbidity and length of stay. However, duration of surgery and hospital costs were significantly higher in the robotic surgery cohort [20]. The results of our study lay in stark contrast to these studies, showing a clear benefit of robotic surgery in the emergent and urgent management of acute diverticulitis despite the increased hospitalization cost.

In a nationwide sample representing 25% of US inpatient admissions, this PSM analysis reports a 50% reduction in conversion to open surgery rates with robotic surgery compared to laparoscopic surgery in both emergent and urgent settings. Although conversion to open surgery should not be interpreted as an intraoperative complication, it is important to report it due to its close association with increased postoperative morbidity, hospitalization cost, patients’ quality of life, and higher rates of ostomy creation during the index operation [2, 14]. A meta-analysis by Panin et al. comparing robotic with laparoscopic surgery identified a trend towards lower rates of conversion to open surgery with robotic surgery; however, this was not statistically significant [2]. In a meta-analysis pooling data from 9 studies comparing laparoscopic and robotic surgery in diverticulitis, the authors also identified a lower rate of conversion to open surgery [21]. Our study is one of the largest cohorts in the literature, which may have been better powered to detect a significant difference. The observed differences may be due to enhanced three-dimensional visualization with the robotic camera and the use of wristed instruments, which allow for more precise and careful dissection and suturing. In addition, the robot’s ability to provide better access to more confined spaces, such as the pelvis, could also have played a role. These features can be especially important in non-elective operations, where severe inflammation may affect visualization and tissues are more friable [22, 23].

Moreover, our study identified a benefit of robotic colectomy over open and laparoscopic approaches in reduced rates of anastomotic leaks. While minimally invasive surgery has been shown to have lower anastomotic leaks in the elective patient population, this has not been established before in non-elective procedures [24, 25]. Interestingly, this association was not observed in urgent colectomies, which we defined as procedures performed after hospital days 3–12. Studies in the literature have shown worse outcomes in patients with acute diverticulitis undergoing a delayed surgical intervention, which could potentially be the result of worse inflammatory changes [22]. Although the reasons for the timing of surgery could not be assessed in the current study, the observed rates of anastomotic leaks in the urgent setting in this report were higher than those in the emergent setting.

The reduced rate of ostomy creation among patients undergoing an emergent or urgent robotic colectomy for diverticulitis compared to laparoscopic resection is also an important outcome. Prior single-institution studies had failed to identify such a difference, perhaps due to smaller sample sizes [26]. In a recent study by Le et al., the authors compared patients with acute diverticulitis who underwent an emergent open vs combined minimally invasive laparoscopic or robotic colectomy. The authors showed that a minimally invasive approach was independently associated with lower rates of ostomy creation at the index operation [6]. The financial and quality of life implications of avoiding an ostomy are significant [6]. Although the exact reasons for this association remain unknown, potential explanations may include wristed instruments, better visualization of tissue planes, and possible selection bias may also exist in the robotic group towards surgeons with higher expertise in minimally invasive colorectal surgery. Of note, our study inclusion years (2019–2023) did not cross over the landmark randomized controlled trial published in August 2023 comparing colectomy and end stoma creation to colectomy with primary anastomosis (with or without diversion) in adults with Hinchey class III or IV acute diverticulitis, which showed equivalent post-operative outcomes with lower 12-month stoma-free survival in the Hartmann’s cohort [27].

Our study also reinforces findings in the literature of lower inpatient mortality and a higher likelihood of discharge home with minimally invasive colectomies for diverticulitis compared with open surgery [6]. In our analyses, in contrast to the study by Monzavi et al., patients undergoing a minimally invasive colectomy had lower 30-day readmission rates when compared to open surgery [5]. Of note, from our comparison groups, in terms of inpatient mortality, final disposition, and 30-day readmission rates no added benefit was offered with robotic over laparoscopic surgery.

Over the past 1–2 decades, there has been a rapid expansion in the use of robotic surgery in emergency general surgery. However, the clinical benefits of robotic surgery remain debated [28]. As a response, the World Society of Emergency Surgery published in 2021 an expert consensus position paper [29]. In this report, the authors emphasized the lack of high-level evidence showing the benefits of robotic surgery over other modalities, in order to justify the longer operating room times and higher financial burden to the institution and the patient. In addition, there was consensus on the need for proper patient selection, prioritizing open surgery for unstable patients, and using robotic technology only by highly trained surgeons with experienced, trained ancillary staff [29]. While prior, mostly smaller, studies in the literature have had conflicting results regarding the use of robotic surgery in emergent/urgent colectomies, our study shows compelling benefits of the robotic technology over other approaches (open and laparoscopic modalities) in the management of acute diverticulitis in the acute care setting. These demonstrated benefits make the necessary financial investments to obtain the technology, train surgeons and staff, and maintain a robotic training curriculum for the trainees worth pursuing [23].

The use of a dataset that covers approximately 25% of inpatient hospital admissions nationwide, across a variety of geographic locations, types of surgeons, and practice settings, allows for good generalizability of our results. However, the results of our study should be interpreted in light of the following limitations. First, although we used propensity score matching to account for all available covariates, our study is predisposed to patient selection bias. Despite the robust propensity score-matching analysis, potential selection bias may persist due to factors not captured in the database such as Hinchey classification, patients’ surgical history, case complexity, surgeon preferences, intra-operative decision making, and institutional robotic team protocols and expertise. Although we attempted to adjust for illness severity by incorporating ICU admission occurring prior to or within 1 day after surgery and the presence of peritoneal abscess as proxies, unmeasured differences in clinical status may remain. Second, due to under coding of robotic ICD/CPT codes, there is a potential risk of misclassification between robotic and laparoscopic cases. Third, administrative datasets have inherent limitations, and there is potential for miscoding and failure to capture additional relevant confounders (e.g., robotic surgery converted to laparoscopic surgery). Fourth, our study period crosses over the COVID-19 pandemic, which has been linked to higher rates of non-operative management for acute diverticulitis, different access minimally invasive platforms, and longer delays in presentation [30].

These limitations notwithstanding, our study has important implications regarding the expansion of robotic surgery in the acute care setting for the management of diverticulitis. In the current era, robotic surgery is often used interchangeably with laparoscopic surgery at the operating surgeon's discretion. Studies to expand our knowledge of which procedures are associated with improved patient outcomes when robotic surgery is used are pressing. Based on our findings, robotic approaches have better patient outcomes to laparoscopic modalities in the urgent/emergent management of acute diverticulitis. As a result, the financial investment required to obtain and maintain robotic platforms as well as train the healthcare providers to use it and the longer operative times can be justified in this setting [2]. Appropriate patient selection remains of utmost importance, and conventional open surgery is recommended in patients who are hemodynamically unstable [29]. With the need to teach fully trained surgeons being the largest obstacle in the dissemination of robotic technology, many industry-led curricula have been developed. However, these curricula are not trainee focused, and early studies have highlighted the resulting decreased intraoperative responsibility and autonomy afforded to trainees [11, 31]. With the wide adoption of robotic surgery, training program led curricula focused on the trainees are paramount. These curricula should be focused on a stepwise progression of resident graduated autonomy with ongoing milestone-based evaluations by faculty [32].

## Conclusions

Findings from this retrospective cohort study indicate that robotic surgery is a safe modality with improved outcomes in the management of patients with acute diverticulitis requiring an urgent or emergent operation. The robotic platform is expanding rapidly within the emergency general surgery patient population. The World Society of Emergency Surgery position paper presents a unique opportunity for additional research to further explore areas where the robotic modality may improve patient outcomes.

## Supplementary Information

Below is the link to the electronic supplementary material.

Supplementary Material 1

Supplementary Material 2

Supplementary Material 3

## Data Availability

The data that support the findings of this study are available from Premier Inc (https://offers.pinc-ai.com/Premier-Healthcare-Database-Download.html) but restrictions apply to the availability of these data, which were used under license for the current study, and so are not publicly available. Data are however available from the authors upon reasonable request and with permission of Premier Inc.

## Abbreviations

PHD: Premier healthcare dataset
HIPAA: Health insurance portability and accountability act
IRB: Institutional review board
ICD-10: International classification of diseases, 10th revision
PCS: Procedure coding system
CPT: Current procedural terminology
BMI: Body mass index
CCI: Charlson comorbidity index
SSI: Surgical site infection
OR: Operating room
SMD: Standardized mean differences
