# Supplementary material for: Non-elective colectomy for diverticulitis in the U.S.: a retrospective comparison of robotic, laparoscopic, and open approaches
Source: World J Emerg Surg. 2026 May 14;21:41. doi: 10.1186/s13017-026-00700-3 (PMC13347878; doi:10.1186/s13017-026-00700-3)
Supplement: Supplementary file 1 — Supplementary Material 1 [file 13017_2026_700_MOESM1_ESM.docx]

**Supplementary Tables**

Supplementary Table 1: Procedure and diagnosis codes

|  | Codes |
| --- | --- |
| Left/Sigmoid colectomy | ICD-10-PCS: 0DBN0ZZ, 0DBN4ZZ, 0DTN0ZZ, 0DTN4ZZ, 0DBG0ZZ, 0DBG4ZZ, 0DBM0ZZ, 0DBM4ZZ, 0DTG0ZZ, 0DTG4ZZ, 0DTM0ZZ, 0DTM4ZZ |
| Conversions to open | ICD-10-CM: Z53.31, Z53.39 |
| Diverticulitis | ICD-10-CM: K57.20, K57.21, K57.32, K57.33, K57.80, K57.81, K57.92, K57.93 |
| Colorectal cancer and neoplasms | ICD-10-CM: C18.0, C18.1, C18.2, C18.3, C18.4, C18.5, C18.6, C18.7, C18.8, C18.9, C19, C19, C20, C21.0, C21.1, C21.2, C21.8, C77.2, C78.5, C7A.020, C7A.021, C7A.022, C7A.023, C7A.024, C7A.025, C7A.026, C7A.029, D01.0, D12.0, D12.1, D12.2, D12.3, D12.4, D12.5, D12.6, D12.7, D12.8, D12.9, D3A.020, D3A.021, D3A.022, D3A.023, D3A.024, D3A.025, D3A.026, D3A.029, Z85.038 |
| Obese | ICD-10-CM: E66.01, E66.09, E66.2, E66.9, Z68.30, Z68.31, Z68.32, Z68.33, Z68.34, Z68.35, Z68.36, Z68.37, Z68.38, Z68.39, Z68.41, Z68.42, Z68.43, Z68.44, Z68.45 |
| Adhesions | ICD-10-PCS: 0DN84ZZ, 0DNE0ZZ, 0DNE3ZZ, 0DNE4ZZ, 0DNJ0ZZ, 0DNJ3ZZ, 0DNJ4ZZ, 0DNS0ZZ, 0DNS3ZZ, 0DNS4ZZ, 0DNT0ZZ, 0DNT3ZZ, 0DNT4ZZ, 0DNU0ZZ, 0DNU3ZZ, 0DNU4ZZ, 0DNV0ZZ, 0DNV3ZZ, 0DNV4ZZ, 0DNW0ZZ, 0DNW3ZZ, 0DNW4ZZ, 0FN00ZZ, 0FN03ZZ, 0FN04ZZ, 0FN40ZZ, 0FN43ZZ, 0FN44ZZ, 0FN48ZZ, 0FN50ZZ, 0FN53ZZ, 0FN54ZZ, 0 FN57ZZ, 0FN58ZZ, 0FN60ZZ, 0FN63ZZ, 0FN64ZZ, 0FN67ZZ, 0FN68ZZ, 0FN70ZZ, 0FN73ZZ, 0FN74ZZ, 0FN77ZZ, 0FN78ZZ, 0FN80ZZ, 0FN83ZZ, 0FN84ZZ, 0FN87ZZ, 0FN88ZZ, 0FN90ZZ, 0FN93ZZ, 0FN94ZZ, 0FN97ZZ, 0FN98ZZ, 0FNG0ZZ, 0FNG3ZZ, 0FNG4ZZ, 0FNG8ZZ, 0TN00ZZ, 0TN03ZZ, 0TN04ZZ, 0TN07ZZ, 0TN08ZZ, 0TN10ZZ, 0TN13ZZ, 0TN14ZZ, 0TN17ZZ, 0TN18ZZ, 0TN60ZZ, 0TN63ZZ, 0TN64ZZ, 0TN70ZZ, 0TN73ZZ, 0TN74ZZ, 0TNB0ZZ, 0TNB3ZZ, 0TNB4ZZ, 0TNB7ZZ, 0TNB8ZZ, 0TNC0ZZ, 0TNC3ZZ, 0TNC4ZZ, 0TNC7ZZ, 0TNC8ZZ, 0UN00ZZ, 0UN03ZZ, 0UN04ZZ, 0UN08ZZ, 0UN10ZZ, 0UN13ZZ, 0UN14ZZ, 0UN18ZZ, 0UN20ZZ, 0UN23ZZ, 0UN24ZZ, 0UN28ZZ, 0UN50ZZ, 0UN53ZZ, 0UN54ZZ, 0UN57ZZ, 0UN58ZZ, 0UN60ZZ, 0UN63ZZ, 0UN64ZZ, 0UN67ZZ, 0UN68ZZ, 0UN70ZZ, 0UN73ZZ, 0UN74ZZ, 0UN77ZZ, 0UN78ZZ, 0UN90ZZ, 0UN93ZZ, 0UN94ZZ, 0UN97ZZ, 0UN98ZZ, 0UNG7ZZ, 0UNG8ZZ, 0UNGXZZ  CPT: 44005, 44180  ICD-10-CM: K56.5, K66.0, N73.6, N99.4, Q43.3 |
| Peritoneal abscess | ICD-10-CM: K63.2, K65.0, K65.1, K65.2, K65.8, K65.9 |
| Colostomy/ Ileostomy | ICD-10-PCS: 0D1H0Z4, 0D1H4Z4, 0D1H8Z4, 0D1K0Z4, 0D1K4Z4, 0D1K8Z4, 0D1L0Z4, 0D1L4Z4, 0D1L8Z4, 0D1N0Z4, 0D1N4Z4, 0D1N8Z4, 0D1M074, 0D1M0J4, 0D1M0K4, 0D1M0Z4, 0D1M3J4, 0D1M474, 0D1M4J4, 0D1M4K4, 0D1M4Z4, 0D1M874, 0D1M8J4, 0D1M8K4, 0D1M8Z4, 0D1N074, 0D1N0J4, 0D1N0K4, 0D1N3J4, 0D1N474, 0D1N4J4, 0D1N4K4, 0D1N874, 0D1N8J4, 0D1N8K4, 0D1L074, 0D1L0J4, 0D1L0K4, 0D1L3J4, 0D1L474, 0D1L4J4, 0D1L4K4, 0D1L874, 0D1L8J4, 0D1L8K4, 0D190Z4, 0D194Z4, 0D198Z4, 0D1A0Z4, 0D1A4Z4, 0D1A8Z4, 0D1B8Z4, 0D1B4Z4, 0D1B0Z4 |
| Anastomotic leak | ICD-10-CM: K31.6, K63.2, N32.1, T81.83XA, Y83.2, K91.81, K91.89, K65.0 |
| Bleeding | ICD-10-CM: D62, D69.9, K91.840, K91.841, K91.870, K91.871, R58, K92.1, K92.2, K91.61, K91.62 |
| Blood transfusion | ICD-10-PCS: 30233H0, 30233H1, 30233K1, 30233L1, 30233M1, 30233N0, 30233N1, 30233P1, 30233R1, 30243H0, 30243H1, 30243J1, 30243K1, 30243L1, 30243M1, 30243N0, 30243N1, 30243P1, 30243R1, 30253H0, 30253H1, 30253K1, 30253L1, 30253M1, 30253N0, 30253N1, 30253P1, 30253Q1, 30253R1, 30253T1, 30263H0, 30263H1, 30263K0, 30263K1, 30263L0, 30263L1, 30263M1, 30263N0, 30263N1, 30263P0, 30263P1, 30263R0, 30263R1  CPT: 36430 |
| Ileus | ICD-10-CM: K56.0, K56.7, K91.30, K91.31 |
| Surgical site infections | ICD-10-CM: K65.1, K65.2, K65.3, K65.8, K65.9, K67, K68.11, K68.19, K68.9, L03.319, L08.89, L08.9, T85.79XA, K63.0, K68.11, T81.40XA, T81.41XA, T81.42XA, T81.43XA, T81.49XA, T81.40XD, T81.40XS, T81.41XD, T81.41XS, T81.42XD, T81.42XS, T81.43XD, T81.43XS, T81.49XD, T81.49XS |
| Sepsis | ICD-10-CM: A40.0, A40.1, A40.3, A40.8, A40.9, A41.01, A41.02, A41.1, A41.2, A41.3, A41.4, A41.50, A41.51, A41.52, A41.53, A41.59, A41.81, A41.89, A41.9, A42.7, R65.10, R65.20, R65.21, R78.81, T81.44XA, T81.44XD, T81.44XS |
| Bowel obstruction | ICD-10-CM: K56.5, K56.50, K56.51, K56.52, K56.60, K56.600, K56.601, K56.609, K56.69, K56.690, K56.691, K56.699 |

Supplementary Table 2: Baseline Patient Characteristics in Emergent and Urgent Surgery by Surgical Modality

|  | **Emergent Surgery** | | | | **Urgent Surgery** | | | |
| --- | --- | --- | --- | --- | --- | --- | --- | --- |
| **Characteristic** | **Open (N = 13,905)** | **Laparoscopic (N = 3,028)** | **Robotic (N = 1,001)** | **p-value** | **Open (N = 6,652)** | **Laparoscopic (N = 2,750)** | **Robotic (N = 1,120)** | **p-value** |
| **Age continuous** |  |  |  | **<0.001** |  |  |  | **<0.001** |
| Mean (SD) | 62.8 (14.2) | 59.5 (14.7) | 58.3 (14.2) |  | 62.1 (14.4) | 59.5 (14.7) | 59.6 (14.8) |  |
| Median (Q1, Q3) | 64 (53, 74) | 61 (49, 70) | 59 (47, 69) |  | 63 (52, 73) | 61 (49, 70) | 61 (49, 71) |  |
| **Age category, n (%)** |  |  |  | **<0.001** |  |  |  | **<0.001** |
| 18 - 44 years | 1,648 (11.9) | 527 (17.4) | 195 (19.5) |  | 887 (13.3) | 484 (17.6) | 204 (18.2) |  |
| 45 - 64 years | 5,595 (40.2) | 1,301 (43.0) | 439 (43.9) |  | 2,661 (40.0) | 1,210 (44.0) | 462 (41.3) |  |
| 65+ years | 6,662 (47.9) | 1,200 (39.6) | 367 (36.7) |  | 3,104 (46.7) | 1,056 (38.4) | 454 (40.5) |  |
| **Gender, n (%)** |  |  |  | 0.27 |  |  |  | 0.070 |
| Female | 7,347 (52.8) | 1,589 (52.5) | 554 (55.3) |  | 3,697 (55.6) | 1,499 (54.5) | 656 (58.6) |  |
| Male | 6,558 (47.2) | 1,439 (47.5) | 447 (44.7) |  | 2,955 (44.4) | 1,251 (45.5) | 464 (41.4) |  |
| **Hispanic Ethnicity, n (%)** |  |  |  | **<0.001** |  |  |  | **<0.001** |
| Yes | 1,176 (8.5) | 382 (12.6) | 130 (13.0) |  | 681 (10.2) | 398 (14.5) | 204 (18.2) |  |
| No | 11,336 (81.5) | 2,356 (77.8) | 787 (78.6) |  | 5,262 (79.1) | 2,066 (75.1) | 817 (72.9) |  |
| Unknown | 1,393 (10.0) | 290 (9.6) | 84 (8.4) |  | 709 (10.7) | 286 (10.4) | 99 (8.8) |  |
| **Race, n (%)** |  |  |  | **<0.001** |  |  |  | **<0.001** |
| White | 11,808 (84.9) | 2,470 (81.6) | 811 (81.0) |  | 5,470 (82.2) | 2,187 (79.5) | 891 (79.6) |  |
| Black | 972 (7.0) | 218 (7.2) | 85 (8.5) |  | 614 (9.2) | 247 (9.0) | 108 (9.6) |  |
| Other | 719 (5.2) | 204 (6.7) | 70 (7.0) |  | 373 (5.6) | 210 (7.6) | 85 (7.6) |  |
| Unknown | 406 (2.9) | 136 (4.5) | 35 (3.5) |  | 195 (2.9) | 106 (3.9) | 36 (3.2) |  |
| **Charlson Comorbidity Index, n (%)** |  |  |  | **<0.001** |  |  |  | **<0.001** |
| CCI = 0 | 6,276 (45.1) | 1,673 (55.3) | 584 (58.3) |  | 2,895 (43.5) | 1,416 (51.5) | 546 (48.8) |  |
| CCI = 1 | 3,282 (23.6) | 714 (23.6) | 243 (24.3) |  | 1,654 (24.9) | 657 (23.9) | 298 (26.6) |  |
| CCI >= 2 | 4,347 (31.3) | 641 (21.2) | 174 (17.4) |  | 2,103 (31.6) | 677 (24.6) | 276 (24.6) |  |
| **BMI category, n (%)** |  |  |  | 0.82 |  |  |  | 0.089 |
| Not Obese | 10,199 (73.3) | 2,205 (72.8) | 730 (72.9) |  | 4,635 (69.7) | 1,865 (67.8) | 753 (67.2) |  |
| Obese | 3,706 (26.7) | 823 (27.2) | 271 (27.1) |  | 2,017 (30.3) | 885 (32.2) | 367 (32.8) |  |
| **Current or recurrent smoker, n (%)** | 6,404 (46.1) | 1,307 (43.2) | 422 (42.2) | **0.002** | 3,258 (49.0) | 1,317 (47.9) | 517 (46.2) | 0.18 |
| **Adhesions, n (%)** | 1,611 (11.6) | 507 (16.7) | 199 (19.9) | **<0.001** | 1,040 (15.6) | 612 (22.3) | 300 (26.8) | **<0.001** |
| **Peritoneal abscess, n (%)** | 7,609 (54.7) | 1,307 (43.2) | 290 (29.0) | **<0.001** | 2,361 (35.5) | 796 (28.9) | 317 (28.3) | **<0.001** |
| **ICU admission before, during or the day after surgery, n (%)** | 4,843 (34.8) | 504 (16.6) | 66 (6.6) | **<0.001** | 1,702 (25.6) | 289 (10.5) | 106 (9.5) | **<0.001** |
| **Year of admission, n (%)** |  |  |  | **<0.001** |  |  |  | **<0.001** |
| 2019 | 3,194 (23.0) | 694 (22.9) | 124 (12.4) |  | 1,517 (22.8) | 629 (22.9) | 154 (13.8) |  |
| 2020 | 2,775 (20.0) | 612 (20.2) | 159 (15.9) |  | 1,400 (21.0) | 584 (21.2) | 180 (16.1) |  |
| 2021 | 2,854 (20.5) | 641 (21.2) | 194 (19.4) |  | 1,371 (20.6) | 574 (20.9) | 202 (18.0) |  |
| 2022 | 2,804 (20.2) | 587 (19.4) | 251 (25.1) |  | 1,304 (19.6) | 504 (18.3) | 242 (21.6) |  |
| 2023 | 2,278 (16.4) | 494 (16.3) | 273 (27.3) |  | 1,060 (15.9) | 459 (16.7) | 342 (30.5) |  |
| **Hospital location, n (%)** |  |  |  | **<0.001** |  |  |  | **<0.001** |
| Urban | 11,983 (86.2) | 2,661 (87.9) | 927 (92.6) |  | 5,844 (87.9) | 2,459 (89.4) | 1,044 (93.2) |  |
| Rural | 1,922 (13.8) | 367 (12.1) | 74 (7.4) |  | 808 (12.1) | 291 (10.6) | 76 (6.8) |  |
| **Payor, n (%)** |  |  |  | **<0.001** |  |  |  | **<0.001** |
| Medicare | 6,757 (48.6) | 1,216 (40.2) | 385 (38.5) |  | 3,179 (47.8) | 1,106 (40.2) | 470 (42.0) |  |
| Commercial | 4,492 (32.3) | 1,209 (39.9) | 445 (44.5) |  | 2,152 (32.4) | 1,065 (38.7) | 427 (38.1) |  |
| Medicaid | 1,513 (10.9) | 374 (12.4) | 89 (8.9) |  | 722 (10.9) | 321 (11.7) | 112 (10.0) |  |
| Other | 1,143 (8.2) | 229 (7.6) | 82 (8.2) |  | 599 (9.0) | 258 (9.4) | 111 (9.9) |  |
| **Provider Region, n (%)** |  |  |  | **<0.001** |  |  |  | **<0.001** |
| Northeast | 2,156 (15.5) | 574 (19.0) | 112 (11.2) |  | 980 (14.7) | 564 (20.5) | 121 (10.8) |  |
| Midwest | 3,201 (23.0) | 562 (18.6) | 198 (19.8) |  | 1,422 (21.4) | 390 (14.2) | 185 (16.5) |  |
| South | 6,398 (46.0) | 1,434 (47.4) | 548 (54.7) |  | 3,246 (48.8) | 1,378 (50.1) | 646 (57.7) |  |
| West | 2,150 (15.5) | 458 (15.1) | 143 (14.3) |  | 1,004 (15.1) | 418 (15.2) | 168 (15.0) |  |
| **Hospital number of beds, n (%)** |  |  |  | **<0.001** |  |  |  | **<0.001** |
| 0 to 199 beds | 3,467 (24.9) | 908 (30.0) | 187 (18.7) |  | 1,508 (22.7) | 706 (25.7) | 213 (19.0) |  |
| 200 to 499 beds | 6,689 (48.1) | 1,418 (46.8) | 563 (56.2) |  | 3,331 (50.1) | 1,315 (47.8) | 582 (52.0) |  |
| 500+ beds | 3,749 (27.0) | 702 (23.2) | 251 (25.1) |  | 1,813 (27.3) | 729 (26.5) | 325 (29.0) |  |
| **Academic institution, n (%)** | 6,251 (45.0) | 1,286 (42.5) | 392 (39.2) | **<0.001** | 2,928 (44.0) | 1,200 (43.6) | 473 (42.2) | 0.53 |
| **Hospital volume, n (%)** |  |  |  | **<0.001** |  |  |  | **<0.001** |
| Low Volume (0 - 67) | 6,383 (45.9) | 1,370 (45.2) | 327 (32.7) |  | 2,873 (43.2) | 1,043 (37.9) | 381 (34.0) |  |
| Medium Volume (68 - 140) | 4,294 (30.9) | 968 (32.0) | 382 (38.2) |  | 2,260 (34.0) | 1,003 (36.5) | 464 (41.4) |  |
| High Volume (141 - 425) | 3,228 (23.2) | 690 (22.8) | 292 (29.2) |  | 1,519 (22.8) | 704 (25.6) | 275 (24.6) |  |
| **Surgeon volume, n (%)** |  |  |  | **<0.001** |  |  |  | **<0.001** |
| Low Volume (0 - 3) | 6,592 (47.4) | 1,538 (50.8) | 285 (28.5) |  | 2,695 (40.5) | 1,238 (45.0) | 346 (30.9) |  |
| Medium Volume (4 - 10) | 5,165 (37.1) | 877 (29.0) | 269 (26.9) |  | 2,560 (38.5) | 794 (28.9) | 299 (26.7) |  |
| High Volume (11 - 167) | 2,148 (15.4) | 613 (20.2) | 447 (44.7) |  | 1,397 (21.0) | 718 (26.1) | 475 (42.4) |  |

*Abbreviations: SD: Standard Deviation; CCI: Charlson Comorbidity Index; BMI: Body Mass Index; ICU: Intensive Care Unit*
