# Supplementary material for: Non-elective colectomy for diverticulitis in the U.S.: a retrospective comparison of robotic, laparoscopic, and open approaches
Source: World J Emerg Surg. 2026 May 14;21:41. doi: 10.1186/s13017-026-00700-3 (PMC13347878; doi:10.1186/s13017-026-00700-3)

Supplementary Figure 2: Standardized Mean Differences plot for urgent cases: (a) Open vs Laparoscopic surgery, (b) Open vs Robotic surgery, and (c) Laparoscopic vs Robotic surgery.


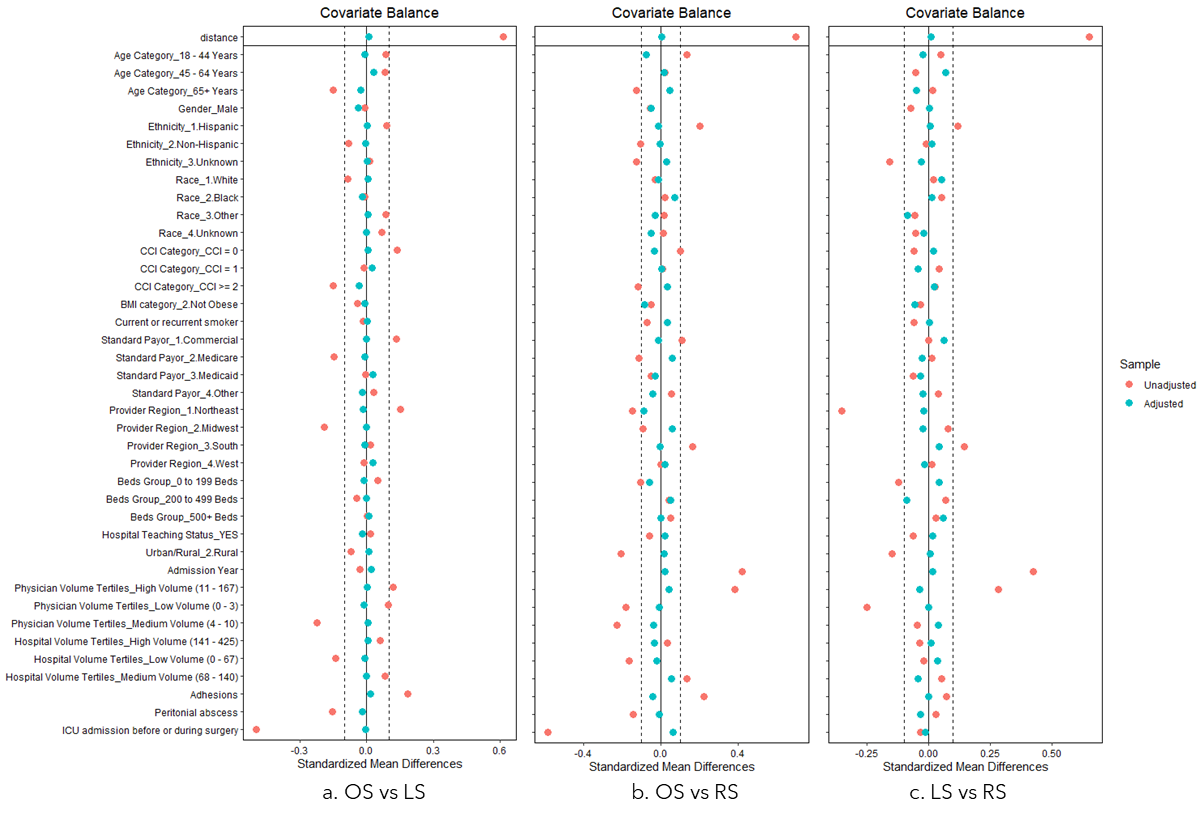

Supplement: Supplementary file 3 — Supplementary Material 3 [file 13017_2026_700_MOESM3_ESM.docx]
